# Effects of Mowing on Methane Uptake in a Semiarid Grassland in Northern China

**DOI:** 10.1371/journal.pone.0035952

**Published:** 2012-04-25

**Authors:** Lihua Zhang, Dufa Guo, Shuli Niu, Changhui Wang, Changliang Shao, Linghao Li

**Affiliations:** 1 State Key Laboratory of Vegetation and Environmental Change, Institute of Botany, Chinese Academy of Sciences, Beijing, China; 2 Shandong Normal University, Jinan, China; USDA-ARS, United States of America

## Abstract

**Background:**

Mowing is a widely adopted management practice for the semiarid steppe in China and affects CH_4_ exchange. However, the magnitude and the underlying mechanisms for CH_4_ uptake in response to mowing remain uncertain.

**Methodology/Principal Findings:**

In two consecutive growing seasons, we measured the effect of mowing on CH_4_ uptake in a steppe community. Vegetation was mowed to 2 cm (M2), 5 cm (M5), 10 cm (M10), 15 cm (M15) above soil surface, respectively, and control was set as non-mowing (NM). Compared with control, CH_4_ uptake was substantially enhanced at almost all the mowing treatments except for M15 plots of 2009. CH_4_ uptake was significantly correlated with soil microbial biomass carbon, microbial biomass nitrogen, and soil moisture. Mowing affects CH_4_ uptake primarily through its effect on some biotic factors, such as net primary productivity, soil microbial C\N supply and soil microbial activities, while soil temperature and moisture were less important.

**Conclusions/Significance:**

This study found that mowing affects the fluxes of CH_4_ in the semiarid temperate steppe of north China.

## Introduction

Methane (CH_4_) is an important greenhouse gas and plays an important role in the global carbon (C) cycle [1]. It has a potent global warming potential (i.e. 25-fold higher than carbon dioxide in mass at a 100-year time horizon [2]) and is increasing at an annual rate of 1% in the atmosphere due to anthropogenic activities [3].

Arid and semiarid grasslands have been considered to be sinks for atmospheric CH_4_ [4], [5]. Recent studies demonstrated that human activities have greatly altered the strength of CH_4_ uptake in grasslands and may affect the global CH_4_ budget [6]–[9]. Mowing, an important human practice in the Eurasian steppe management, has various effects on this semiarid grassland ecosystem [10]–[13], including changes to CH_4_ uptake. Discerning the effect of mowing on CH_4_ fluxes is especially important because mowing is increasingly being used as a method to collect forage and feed livestock relative to traditional grazing practices [14]. Removal of biomass by mowing may affect CH_4_ uptake due to concurrent changes in nutrients for soil microbial growth [15]–[17]. In addition, mowing can alter availability of light to plants [18], soil surface temperature, and moisture [19] that affect CH_4_ production and consumption. However, the magnitude and underlying mechanisms of CH_4_ uptake in response to mowing remain uncertain.

In semiarid grasslands of Inner Mongolia, grazing is another important management practice. Previous studies report that grazing tended to reduce CH_4_ uptake in some grassland ecosystems [20]–[27]. It is further predicted that if the effect of grazing is taken into account, the steppe ecosystem would become a CH_4_ source [28], [29]. In contrast to grazing, mowing has the potential to increase the capacity of the system to function as a CH_4_ sink. We hypothesize that mowing tends to facilitate CH_4_ uptake in grassland ecosystems, because diminished soil inorganic N caused by mowing would result in CH_4_ oxidation [10]. However, there is no direct experimental evidence to support this hypothesis. In addition, it is not clear whether soil feedbacks, especially those in combination with aboveground or abiotic mechanisms, contribute to the changes in CH_4_ uptake in mowed grasslands. Therefore, a better understanding of the magnitude and the underlying mechanisms for CH_4_ exchanges in response to mowing is essential to accurately assess the CH_4_ sink-source functions of Eurasian grasslands in the global carbon budget [1].

The objectives of this study were: (1) to examine the effects of mowing on CH_4_ fluxes in a steppe habitat; (2) to study the effects of mowing on soil chemical and microbial properties; and (3) to determine the optimal mowing height (a surrogate for mowing intensity) that maximizes CH_4_ sink function of the grassland ecosystem.

## Methods

### Site description

The field experiment was conducted in a typical temperate steppe in Duolun County (116°17′E, 42°02′N, 1324 m asl), Inner Mongolia, North China. This area has a continental monsoon climate, being semiarid and temperate in summer. Mean annual temperature is about 2.1°C with monthly mean extreme temperatures of 18.9°C in July and −17.5°C in January. Mean annual precipitation is approximately 385 mm with about 80% occurring from mid-June to late September. The study site's soil is chestnut soil (Chinese classification) or Haplic Calcisols according to the FAO classification, with sand, silt and clay being 62.8%, 20.3% and 16.9% respectively. Mean soil bulk density is 1.31 g cm^−3^ and pH is 7.12 [30]. The dominant plant species are *Artemisia frigida* Willda, *Stipa krylovii* Roshev., *Potentilla acaulis* L., *Cleistogenes squarrosa* (Trin.) Keng., *Allium bidentatum* Fisch. Ex Prokh., and *Agropyron cristatum* (L.) Gaertn.

### Field experimental design

The study site has been fenced to exclude grazing since 2001. From 2003, a 10-ha area in the *Stipa krylovii* community was enclosed, in which mowing (including collection of the hay) plots were established. We used a Latin square design with control and four levels of mowing treatments. Each treatment had five replicates. Twenty-five 10×20 m plots were arranged in a 5×5 matrix. The buffer distance between plots was 4 m. We used mowing height as a surrogate for mowing intensity. Vegetation was mowed at heights of 2 cm (M2), 5 cm (M5), 10 cm (M10),15 cm (M15) above soil surface and the control had non-mowing (NM, about 30 cm). A machine was used to mow the plots once annually in late August since 2003.

### Measurements of CH_4_ flux and above ground plant biomass

The static opaque chamber method [31]–[33] was used to measure CH_4_ flux. One stainless steel base (50×50 cm) was installed into the soil of each plot. The steel base had a groove on top to ensure airtight connection with the chamber (50×50×50 cm) [34]. Two electric fans were installed inside the top of the chamber to mix the air during measurement. Gas samples of 60 mL were collected into syringes with airtight stopcocks at a 10-min interval during the 30 minutes of chamber closure. Simultaneously, air temperature and air pressure in the chamber were measured. Analysis of CH_4_ was conducted using a gas chromatograph (HP 5860, Agilent Technologies), which was equipped with flame ionization detector (FID) using 60–80 mesh 13 XMS column (2 mm inner diameter and 2 m long), with an oven temperature of 55°C. Nitrogen was used as the carrier gas with a flow rate of 30 mL min^−1^, and the CH_4_ flux was determined from changes in the slope of the mixing ratio of four samples taken at 0, 10, 20 and 30 min after chamber closure. Corrections were made for air temperature and pressure. The correlation coefficient of the regression was validated (r^2^≥0.95, n = 4). CH_4_ flux was measured weekly in 2008 from June to September and every two weeks in 2009 from May to September. Meanwhile soil (5 cm) temperature and moisture were measured by the Long-Stem Thermometer 6310 (Made in US) and portable soil moisture measuring kit ML2x (ThetaKit, Delta-T Devices, Cambridge, UK [35]).

Aboveground plant biomass was measured using the harvest method according to Chen [36]. We randomly selected 1 m^2^ square areas from every plot and clipped plant material 1 cm above the ground level.

### Soil sampling and analysis

Soil samples (0–10 cm layer) were collected using soil corers (5 cm diameter) every month during the growing season in 2009. Three soil samples were taken randomly in each plot and mixed evenly. The mixed sample was then divided into two sub-samples, one stored at 4°C for microbial analysis and the other air-dried for soil total C, N and phosphorus (P) analyses. We collected a total of 250 soil samples (5 treatments×5 replicates×2 sub-samples×5 months). Soil microbial biomass carbon (MBC) and nitrogen (MBN) were determined using the chloroform fumigation–extraction method [37] following the protocols described by Liu et al. (2007) [38].

### Statistical analysis

Seasonal mean CH_4_ uptake was calculated from the monthly mean values which were averaged by month. Seasonal cumulative CH_4_ uptake was calculated using a simple linear interpolation, by which the arithmetical mean of the two temporally closest observations was extrapolated to represent the flux of each duration. Differences in seasonal cumulative CH_4_ uptake, average ST, SM, soil MBC, and MBN among treatments were determined by analysis of variance (ANOVA) followed by multiple comparisons (Duncan test). Because the effect of mowing was different between 2008 and 2009, repeated-measures ANOVAs were applied to determine the main and interactive effects of measurement time and mowing treatment on CH_4_ uptake rate, ST, SM, soil MBC and MBN in the two growing seasons, respectively. The linear regression was used to determine the seasonal variation of CH_4_ uptake responses to ST, SM, soil MBC and MBN. Stepwise multiple linear analyses were used to examine post-mowing ecosystem CH_4_ uptake as a function of ST, SM, soil MBC, and MBN. All statistical analyses were conducted with SAS software (SAS Institute Inc., Cary, NC, USA).

## Results

### Effects of mowing on soil temperature and moisture

**Figure 1:**
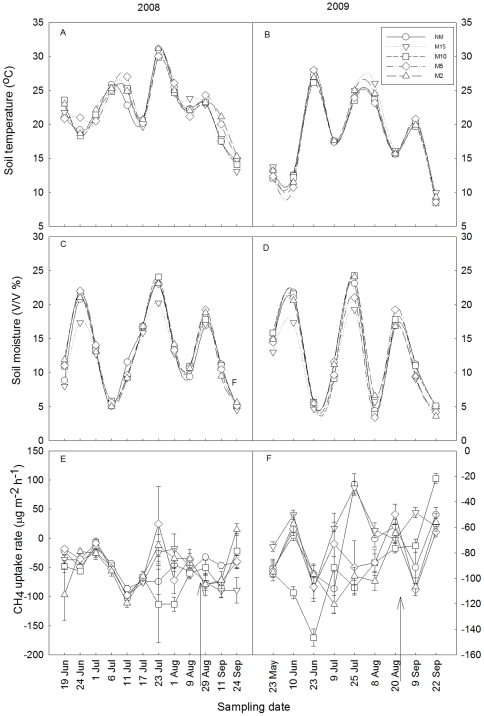
Seasonal variations in soil temperature (A, B) and volumetric soil moisture (C, D) at the soil depth of 0–10 cm, and in fluxes of CH_4_ (E, F) in control and the four mowing treatments in 2008 (left) and 2009 (right); Data are mean ±SE (n = 5). The arrow indicated the mowing date every year.

Soil temperature (ST; Fig. 1 A, B) and soil moisture (SM; Fig. 1 C, D) varied substantially throughout the growing seasons. Soil temperature was relatively low in May and September, while it was higher in July (Fig. 1A, B). Soil moisture was relatively high in July (Fig. 1C, D). Soil temperature was negatively correlated with mowing height (r^2^ = 0.74, p<0.001). Only 15 cm and 2 cm mowing height treatments significantly affected soil temperature (Table 1), whereas no regular correlation or significant effects were found between mowing height and soil moisture. However, there was a significant interactive effect between sampling date and all mowing treatments on soil temperature (p<0.0001) and soil moisture (p<0.0001) (Table 1).

**Table 1:** Results (P values) of repeated measures ANOVAs on the effects of mowing (M), sampling date (D), and their interactions on soil temperature (ST), soil moisture (SM), soil microbial biomass carbon (MBC), soil microbial biomass nitrogen (MBN) and CH_4_ uptake rate in all the mowing treatments.

		ST	SM	MBC	MBN	CH_4_	
						2008	2009
M15	D	<0.0001	<0.0001	0.033	0.0015	<0.0001	<0.0001
	M	0.0127	0.1185	0.1816	0.0051	0.2841	0.1031
	D×M	0.0002	<0.0001	0.2609	0.1322	0.0055	0.0171
M10	D	<0.0001	<0.0001	0.0543	0.5226	0.0366	0.0015
	M	0.9604	0.1231	0.0852	0.2153	0.067	0.0738
	D×M	0.0082	<0.0001	0.4644	0.3332	0.4018	0.0128
M5	D	<0.0001	<0.0001	0.0311	0.3296	0.0306	0.0006
	M	0.1366	0.1745	0.2025	0.5497	0.7462	0.2509
	D×M	0.0293	<0.0001	0.3787	0.2891	0.0984	0.0145
M2	D	<0.0001	<0.0001	0.0221	0.001	0.002	0.0004
	M	0.0033	0.1096	0.3951	0.7815	0.9513	0.1069
	D×M	0.0063	0.0002	0.3019	0.6835	0.0071	0.0142

### Changes in soil microbial carbon and nitrogen

**Figure 2:**
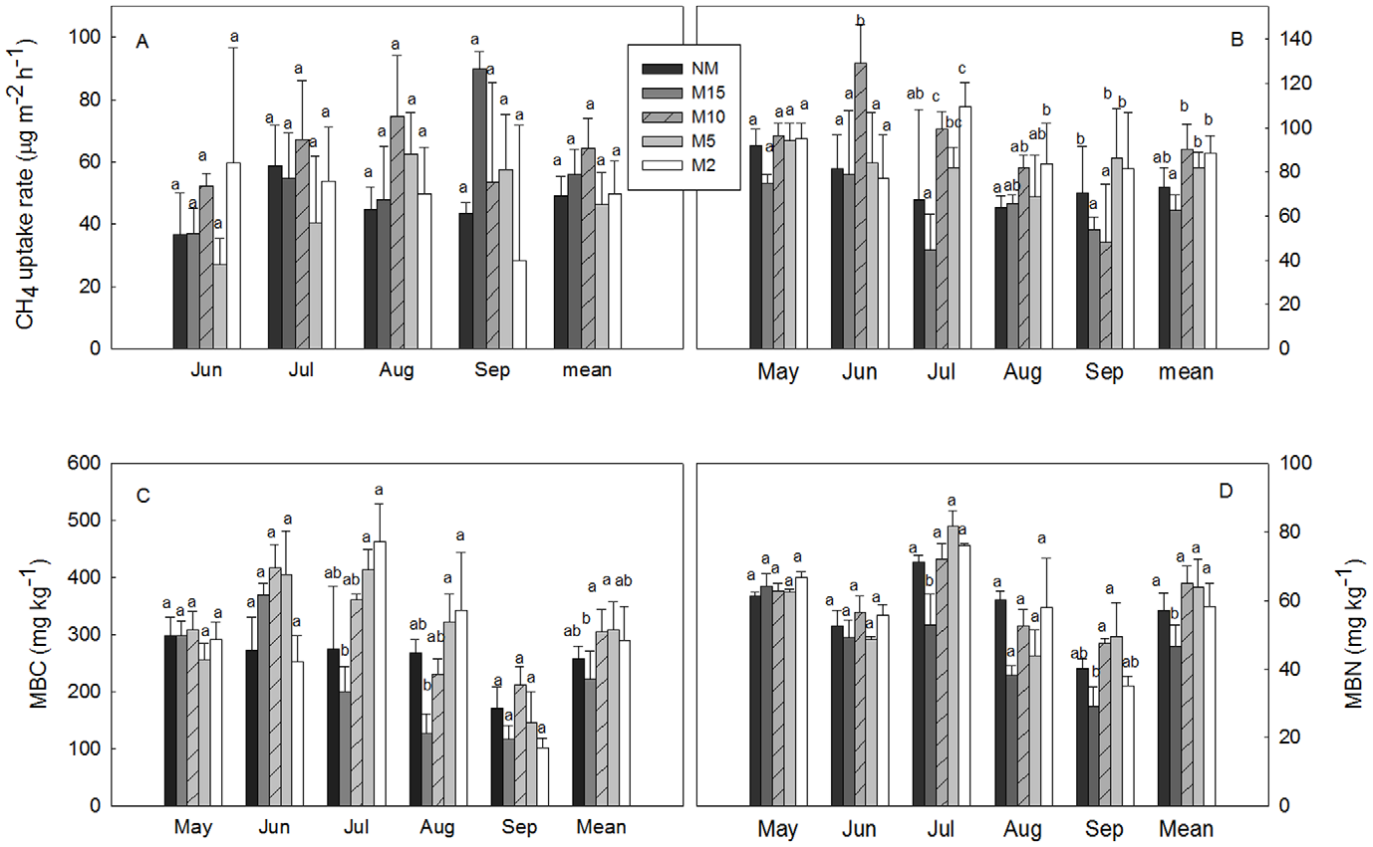
Monthly average CH_4_ uptake in control and different mowing treatments in 2008 (A) and 2009 (B), and effects of mowing on microbial biomass carbon (MBC) (C), microbial biomass nitrogen (MBN) (D). Vertical bars represent the standard error of the means (n = 5). Different letters between columns mean significant difference among treatments at P<0.05.

Both soil microbial biomass carbon and nitrogen (MBC and MBN) showed strong seasonal fluctuations with peak values (for no mowing and all mowing treatments) between June and July 2009 (Fig. 2C, D). Mostly, there was no effect of mowing treatments on MBC or MBN, except a marginally significant effect of one of the mowing treatments (M10) on soil MBC (p = 0.085) and a significant effect of another (M15) on soil MBN (p = 0.005). No significant interactive effects were found between sampling date and mowing on soil MBC and MBN for all the treatments (Table 1). Soil MBC in all the mowing treatments and soil MBN in M15 and M2 were strongly affected by sampling date (p<0.05). Changes in soil MBC and MBN became more evident from May to August; after which they remained almost unchanged (Fig. 2 C, D). Except for M15, other mowing treatments increased the seasonal averaged soil MBC and MBN (Fig. 2 C, D). Compared with control, M10, M5 and M2 enhanced soil MBC by 19.1%, 20% and 12.8%, and soil MBN by 2.0%, 0.2%, 2.0%, respectively. In contrast, the lightest level of mowing (M15) reduced soil MBC by 13.3% and soil MBN by 18.3%, respectively.

### Effects of mowing on methane uptake

There were substantial seasonal variations in CH_4_ uptake for control and the mowing treatments in both 2008 and 2009 (Fig. 1E, F). The greatest CH_4_ emissions were in late July (Fig. 1E, F) during which soil moisture (Fig. 1C, D) and soil temperature (Fig. 1A, B) was also the highest. Inter-annual variations in CH_4_ uptake were also observed.

**Figure 3:**
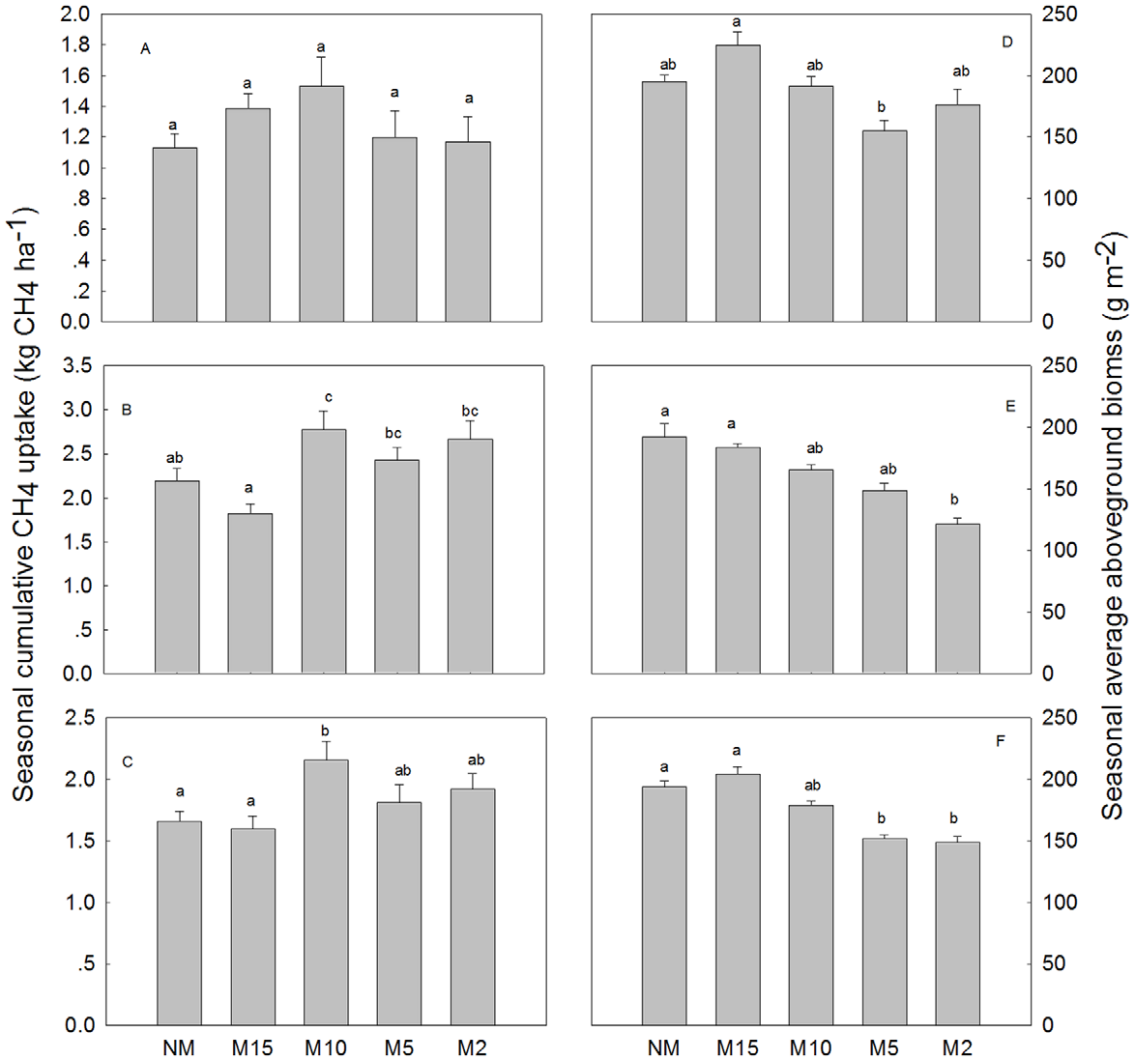
Cumulative methane uptake in 2008 (A), 2009 (B) and the overall of the two growing seasons (C), and net aboveground primary productivity (ANPP) in 2008 (D), 2009 (E) and average of the two seasons (F) in response to mowing intensity. Values represent the mean±SE (n = 5). Different letters between columns mean significant difference among treatments at P<0.05.

Mowing had different effects on the CH_4_ uptake rate at different temporal stages and different treatments (Fig. 2A, B). For instance, during the dry and warm periods during the growing season CH_4_ uptake rates were highest at M10 plots in 2008 and 2009 (Fig. 2A, B). When the seasonal cumulative uptake data in 2008 and 2009 were analyzed separately and collectively using ANOVA multiple comparison analysis, only one mowing treatment (M10) increased CH_4_ uptake relative to the no mowing and the M15 mowing treatment in 2009 (Fig. 3 B) as well as during 2008–2009 (Fig. 3 C). Moreover, there were significant interactive effects of the sampling date and mowing on CH_4_ uptake rate for all treatments in 2009 (p<0.05), and for M15 and M2 in 2008 (Table 1). Generally, the grassland was acting as a CH_4_ sink in the two growing seasons (Fig. 2 A, B; Fig. 3 A–C), and mowing had positive effects on the CH_4_ uptake with intermediate mowing height having the greatest impact.

## Discussion

### Soil temperature and moisture related to methane uptake

**Figure 4:**
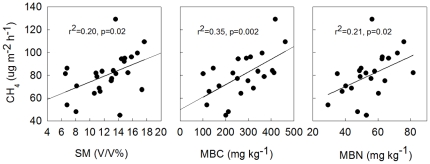
Dependence of seasonal variation in CH_4_ uptake on soil moisture (SM), microbial biomass carbon (MBC) and microbial biomass nitrogen (MBN).

Positive correlations between CH_4_ uptake and soil temperature have been reported in several studies [22], [32], [39]–[41]. However, our results show that no significant correlations between soil temperature and CH_4_ uptake were found during the growing season, but positive correlations between soil moisture and CH_4_ uptake were significant (Fig. 4), which is consistent with that reported by Livesley [42]. Other previous studies also reported that soil moisture associated with soil diffusivity is the major factor controlling CH_4_ uptake rate in the field [8], [41], while soil temperature is just a covariate [43], [44].

**Figure 5:**
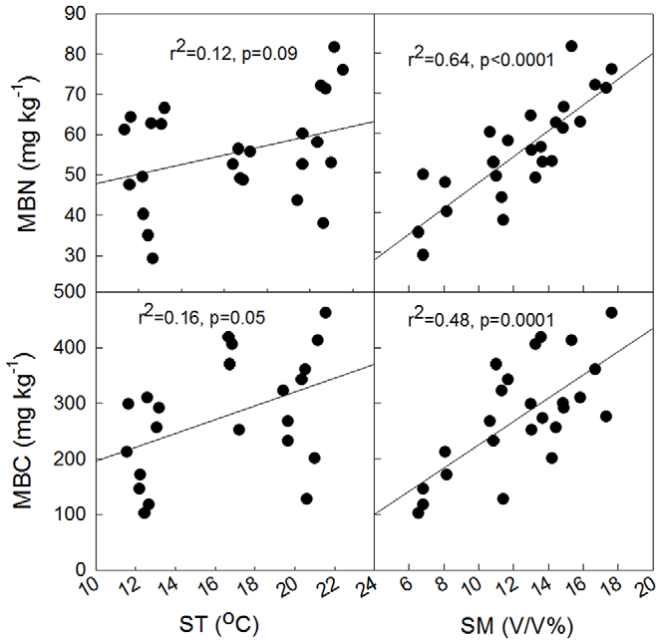
Correlations between soil temperature (ST) and microbial biomass carbon\nitrogen (MBC\MBN), and between soil moisture (SM) and microbial biomass carbon\nitrogen (MBC\MBN).

Further analyses revealed that a combination of soil temperature (ST) and soil moisture (SM) slightly improved the correlation between CH_4_ uptake rate and SM (Y = 61.82−1.30ST+3.21SM, r^2^ = 0.26, p = 0.04), suggesting that SM is the dominant environmental factor controlling CH_4_ uptake in the study area. Previous studies reported that the activity of methanotrophs can be greatly inhibited by small variation in soil moisture [45]. Therefore, CH_4_ oxidation in dry soils is likely to be limited due to low microbial activity occurring during periods of low levels of soil moisture [46]. Similiarly, we found that there were positive relationships between SM and soil MBC\MBN (Fig. 5), and between soil MBC\MBN and CH_4_ uptake rate (Fig. 4).

### Soil microbial carbon and nitrogen associated with methane

Stepwise multiple regression analyses showed that soil MBC and MBN were positively correlated with CH_4_ uptake. Variations in soil MBC and MBN explained 34.9% (p = 0.002) and 20.7% (p = 0.022) of variations in CH_4_ uptake, respectively (Fig. 5). Soil moisture was positively correlated with soil MBC and MBN, explaining 48.4% and 68.3% of variations in soil MBC and MBN, respectively (p<0.0001) (Fig. 5), during the 2009 growing season. When the control and mowing treatments were considered separately, the same correlations between soil MBC, MBN and CH_4_ uptake were observed, and the best correlation was found in M10 treatment.

### Mowing-induced changes in methane uptake

Our results show that effects of mowing on CH_4_ uptake were greatly dependent on the mowing height (Fig. 2 A, B). Moderate mowing heights (M10) enhanced CH_4_ uptake while the tallest mowing height (M15) resulted in less CH_4_ uptake than the M10 height, whereas no significant effects were found for other treatments (Fig. 2 B). Our study helps to illustrate that the effects of mowing on CH_4_ are complex and possibly mediated by: (1) changes to soil moisture; 2) changes to soil C/N supply possibly as a result of altered NPP; and 3) affects on soil microbial C and N.

While soil moisture was positively associated with CH_4_ uptake, mowing treatments generally had no effect on soil moisture except for two mowing treatments (M15, M2) (Table 1). This suggests mowing is affecting CH_4_ by affecting factors other than soil moisture. We observed that there were no apparent differences in standing dead, ground litter and canopy height between mowed and un-mowed plots in the growing seasons. However, light levels of mowing (M15) resulted in lower soil temperature and was associated with changes in community composition such as reduced forbs. This might explain the reduced CH_4_ uptake in M15 (Fig. 2 and 3), since CH_4_ oxidation is likely to be limited due to low microbial activity with reduced soil temperature.

**Figure 6:**
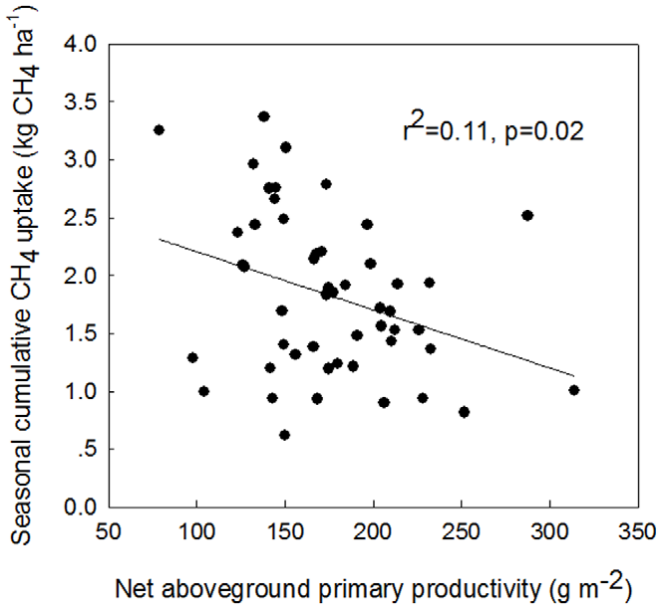
Dependence of seasonal cumulative CH_4_ uptake on the net aboveground primary productivity (ANPP, g m^−2^).

We found CH_4_ uptake was negatively correlated with net above ground primary productivity (ANPP) (Fig. 6). This correlation may be the result of a shift in the intensity of competition between plants and CH_4_ oxidation microbes for soil nutrients, water and other resources. Soil microorganisms are known to respond to alterations in plant-derived C supply [47]. A number of studies reported that changes in soil inorganic N availability [48], due to reduced amounts of C entering into the soil, were responsible for changes in soil CH_4_ oxidation microbial activities [49]. In grassland ecosystems, long-term harvesting by mowing has been shown to divert plant C from soils, posing negative effects on soil microbial populations [50] and forage production (ANPP) [14]. Here light and intermediate mowing (M15, M10) had no effect on ANPP while more intensive mowing treatments (M5, M2) reduced ANPP (Fig. 3 D, E, F). Though mowing had subtle effects on ANPP, these effects correspond with the direct effects of mowing on CH_4_ suggesting a link between ANPP and CH_4_. Similar results have been reported by Whiting and Chanton in a wetland [51].

**Figure 7:**
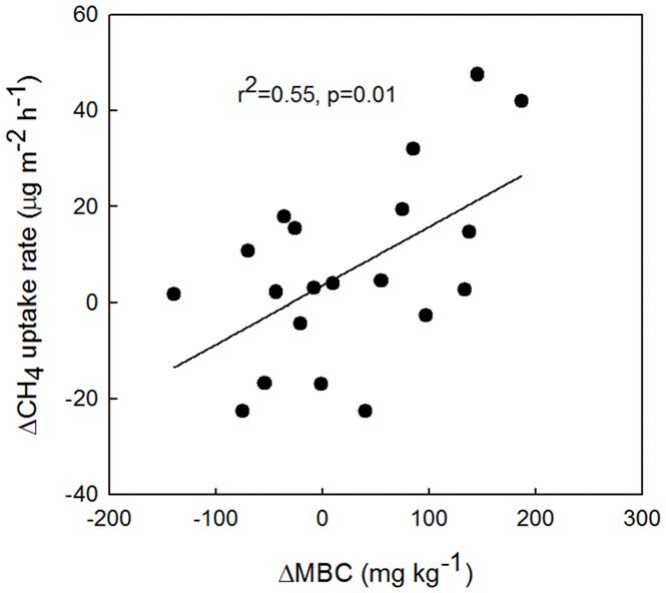
Mowing-induced changes in seasonal mean CH_4_ uptake rate and seasonal mean microbial biomass carbon (MBC).

In our study, mowing-induced increases in CH_4_ uptake may be mediated by changes in MBC and MBN (Fig. 2 C, D and Fig. 7). It has been reported that reduction in inorganic N by mowing resulted in an increase of CH_4_ oxidation [52] and stimulation of root exudation, favoring the microbial activity [53]. Other soil physical environmental factors caused by mowing could be co-responsible. For example, some have observed greater CH_4_ uptake rates in soil cores in New Zealand where type I methanotrophs are dominant [54]. And in our study, the increase in CH_4_ uptake with mowing could also result from changes in methanotrophy community structure and activity [55]. Finally, there are some other factors that can affect the CH_4_ uptake, such as variation of root/shoot ratios [56] and species composition [57] after mowing.

In general, our study demonstrates that moderate mowing can substantially enhance CH_4_ uptake in the semiarid steppe ecosystem. Long-term mowing increased CH_4_ uptake mainly due to its effect on soil biotic factors. 10 cm appeared to be the optimal mowing height. The substantial inter-annual variations in CH_4_ uptake indicate that it is necessary to conduct long-term observations in grasslands in the future to accurately determine the optimal mowing height for enhancing CH_4_ uptake.
